# Exploring blood lipids-immunity associations following HBV vaccination: evidence from a large cross-sectional study

**DOI:** 10.3389/fcimb.2024.1369661

**Published:** 2024-03-08

**Authors:** Qian Yang, Benhua Li, Tiankuo Luan, Xiaoyu Wang, Bixia Duan, Chengcheng Wei, Shi Chen

**Affiliations:** ^1^Clinical Molecular Medicine Testing Center, The First Affiliated Hospital of Chongqing Medical University, Chongqing, China; ^2^Chongqing Key Laboratory of Molecular Oncology and Epigenetics, The First Affiliated Hospital of Chongqing Medical University, Chongqing, China; ^3^Department of Urology, Union Hospital, Tongji Medical College, Huazhong University of Science and Technology, Wuhan, China

**Keywords:** lipid, hepatitis B vaccination, immunity, HDL, HBV - hepatitis B virus

## Abstract

**Introduction:**

Serological responses following hepatitis B vaccination are crucial for preventing hepatitis B (HBV). However, the potential relationship between serum lipid levels and immunity from HBV vaccination remains poorly understood.

**Methods:**

In this study, we conducted an analysis of the National Health and Nutrition Examination Survey (NHANES) data spanning from 2003 to 2016. Multivariable weighted logistic regression models, generalized linear analysis, stratified models, smooth curve fitting, segmentation effect analysis and sensitivity analysis were utilized to assess the relationships.

**Results:**

After adjusting for relevant covariates, we observed that low levels of high-density lipoprotein cholesterol (HDL) were independently linked to a significantly lower seroprotective rate. Compared to HDL levels of ≥ 60 mg/dL, the odds ratios (ORs) for individuals with borderline levels (40-59 mg/dL for men, 50-59 mg/dL for women) and low levels (< 40 mg/dL for men, < 50 mg/dL for women) were 0.83 (95% CI 0.69-0.99) and 0.65 (95% CI 0.56-0.78), respectively. This association was particularly pronounced in individuals aged 40 or older. Conversely, higher levels of the triglyceride to HDL (TG/HDL) ratio (OR, 0.90; 95% CI, 0.84-0.98), total cholesterol to HDL (Chol/HDL) ratio (OR, 0.77; 95% CI, 0.64-0.92), and low-density lipoprotein to HDL (LDL/HDL) ratio (OR, 0.85; 95% CI, 0.76-0.96) were associated with a decreased likelihood of seroprotection.

**Conclusion:**

This study suggests that lipid levels may play a role in modulating the immune response following HBV vaccination.

## Graphical Abstract

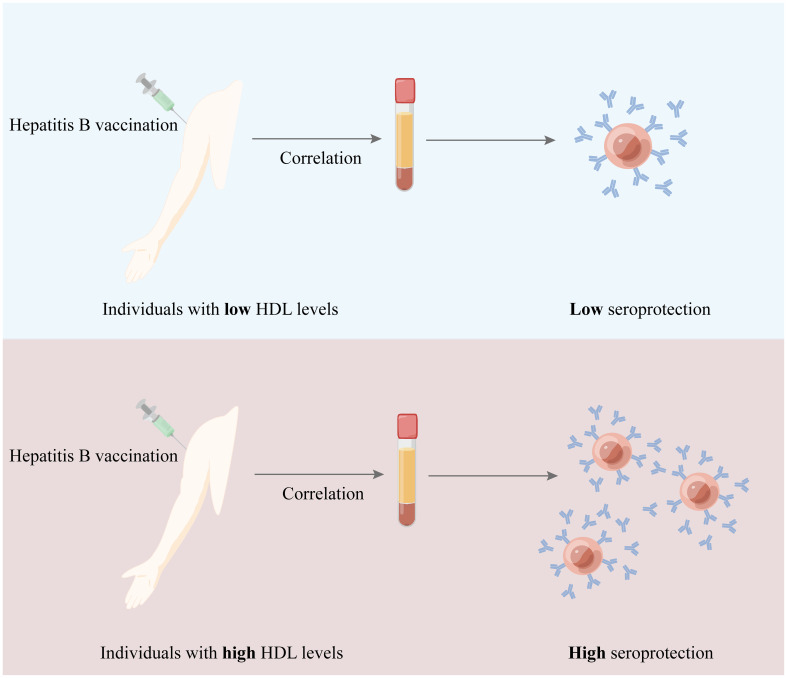

## 1 Introduction

HBV infection can advance to chronic hepatitis B and, in more severe cases, lead to cirrhosis and hepatocellular carcinoma (Global, regional, and national burden of hepatitis B, 1990-2019: a systematic analysis for the Global Burden of Disease Study 2019, 2022). As per a survey by the World Health Organization, an estimated 296 million people globally (3.8%) are chronically infected with HBV, resulting in approximately 820,000 deaths annually (Cui et al., 2023; Jeng et al., 2023). In response to this significant health burden, hepatitis B vaccination, including the administration of a birth dose, has been implemented as a key component of the Immunization Agenda 2030 (IA2030), which is endorsed by the World Health Assembly. Serological protection from vaccination is considered achieved when anti-HBs titers are ≥ 10 mIU/mL. However, despite the demonstrated effectiveness of the hepatitis B vaccine, a subset of individuals fails to attain seroprotection (Muhoza et al., 2021; Chang et al., 2022; El-Sayed and Feld, 2022; Wong et al., 2022). Identifying the factors that influence seroprotection is crucial for enhancing vaccination strategies and reducing the impact of HBV-related diseases. While factors such as age, sex, obesity, smoking, and HIV infection have been reported to affect the immune response to hepatitis B vaccination, additional determinants of seroprotection warrant further investigation (Alavian et al., 2008; Deng et al., 2022; Di Lello et al., 2022; Fonzo et al., 2022; Lian and Morrish, 2022).

Abnormal levels of lipids, including Chol, TG, HDL, and LDL, are associated with various health conditions. Elevated LDL levels are implicated in the development of cardiovascular diseases, while hypertriglyceridemia is linked to nonalcoholic fatty liver disease and acute pancreatitis. The TG/HDL ratio is recognized as a marker for metabolic syndrome (Ryan et al., 2018; Sulaiman, 2020; Pirillo et al., 2021). Lipid metabolism also plays a significant role in modulating immune responses, with implications for T cells, macrophages, dendritic cells, and other immune cell types in diseases such as nonalcoholic fatty liver disease, pancreatic fibrosis, and cancer progression (Yan and Horng, 2020; Liu et al., 2021; Yu et al., 2021; Lim et al., 2022; Zhao et al., 2022). Additionally, lipid-based nanoparticles have been shown to be effective vaccine adjuvants, enhancing antibody responses in vaccines targeting pathogens like SARS-CoV-2, HIV, and HBV (Di Paolo et al., 2010; Bartlett et al., 2020; Lou et al., 2020; Park et al., 2021; Bevers et al., 2022). Despite these findings, the impact of serum lipid levels on immunity following vaccination remains underexplored.

This study aims to investigate the association between serum lipid levels and seroprotection following hepatitis B vaccination, using a large sample of individuals who have received three doses of the vaccine from the National Health and Nutrition Examination Survey (NHANES).

## 2 Materials and methods

### 2.1 Research population

NHANES database is a comprehensive program designed to assess the health and nutritional status of adults and children in the United States. It employs a biennial sampling strategy to select a nationally representative sample of approximately 10,000 individuals through a meticulous sampling process. Participants are randomly chosen to partake in interviews that cover demographic, socioeconomic, dietary, and health-related topics, in addition to undergoing medical, dental, and physiological measurements and laboratory tests.

**Figure 1:**
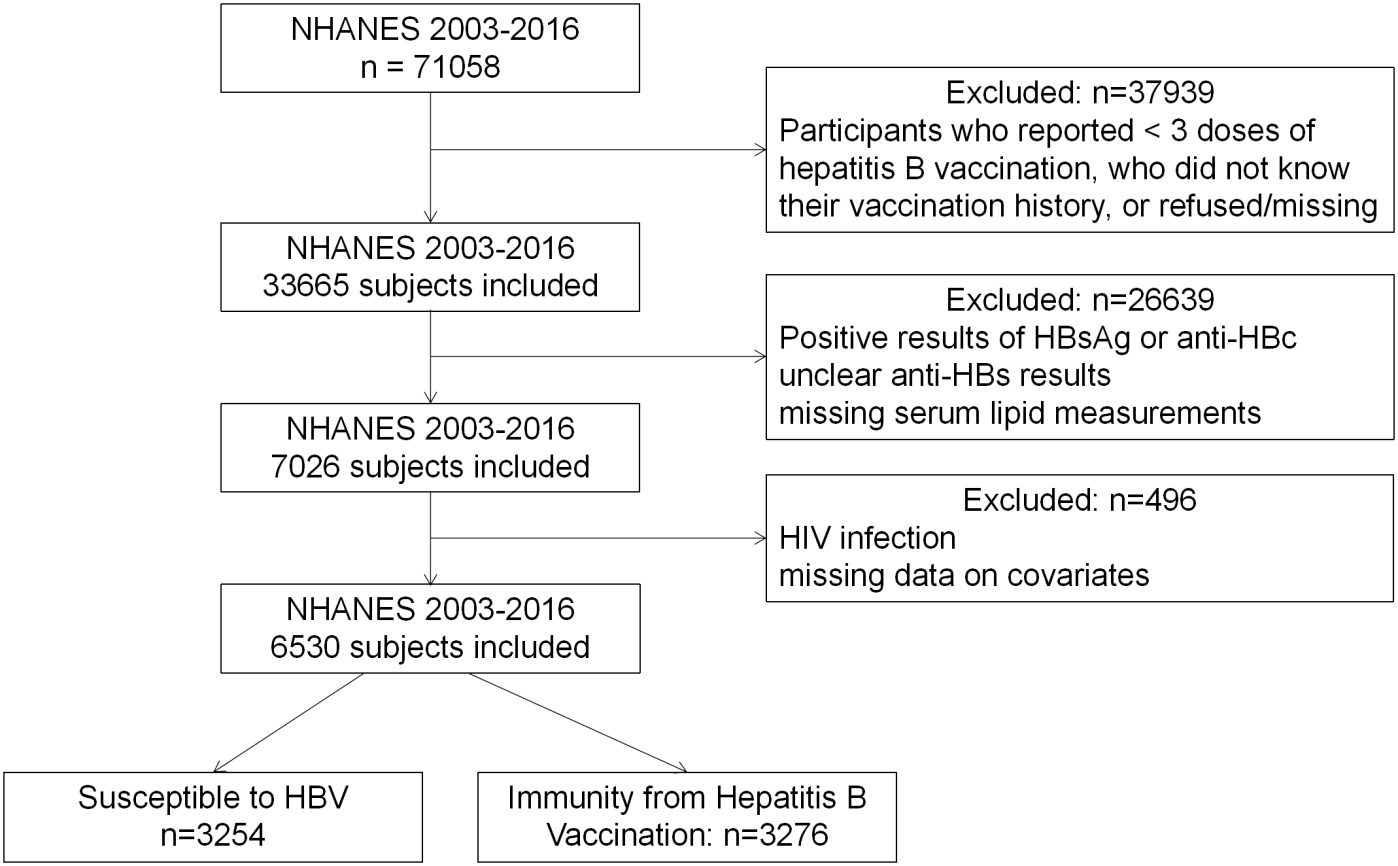
Flow chart of the population included in the final analysis of our study.

For this cross-sectional study, we analyzed data from seven NHANES cycles spanning from 2003 to 2016 (**Figure 1**). Out of the total 71,058 subjects (35,122 men and 35,936 women), 33,665 had completed three doses of the hepatitis B vaccine, and 25,524 had available anti-HBs data. We excluded individuals who tested positive for HBsAg, anti-HBc, or HIV antibodies, indicative of potential immunosuppression. The final study subjects comprised 6,530 unique participants with available data on serum lipids and covariates. This population was further divided into two groups: those with seroprotection against HBV vaccination (n=3,276) and those susceptible to hepatitis B (n=3,254).

### 2.2 Hepatitis B serology assessment

HBV serological markers, including hepatitis B surface antigen (HBsAg), hepatitis B surface antibody (anti-HBs), and hepatitis B core antibody (anti-HBc), were evaluated using the VITROS ECi/ECiQ Immunodiagnostic Systems and VITROS 3600 immunodiagnostic system (Ortho Clinical Diagnostics). The combined assessment of these markers enabled the evaluation of immunity against HBV infection. Individuals with anti-HBs levels equal to or exceeding 10 mIU/mL were considered to have acquired immunity either from vaccination or from the resolution of a previous HBV infection. The presence of HBsAg indicated acute or chronic HBV infection, while anti-HBc positivity suggested previous or ongoing HBV infection. In our study, only participants negative for HBsAg and anti-HBc and positive for anti-HBs were considered to have immunity from vaccination (seroprotection), while those negative for all these markers were classified as nonresponsive after vaccination.

### 2.3 Serum lipid level assessment

In this study, cholesterol (Chol), LDL, HDL, and triglycerides (TG) were measured using the UniCel® DxC 800 Synchron & UniCel® DxC 660i Synchron Access Clinical Systems (Beckman Coulter Diagnostics) or Cobas 6000 Chemistry Analyzer (Roche Diagnostics). Chol and HDL assessments were conducted for participants aged 6 and above, while TG and LDL measurements were taken for those aged 12 and older during the morning session. These measurements were categorized according to clinical guidelines (Christensen et al., 2015). Hyperlipidemia was defined as total cholesterol ≥ 200 mg/dL, triglycerides ≥ 150 mg/dL, LDL ≥ 130 mg/dL, or HDL ≤ 40 mg/dL in men and ≤ 50 mg/dL in women, following the National Cholesterol Education Program (NCEP) guidelines for adults (Executive summary of the third report of the national cholesterol education program (NCEP) expert panel on detection, evaluation, and treatment of high blood cholesterol in adults (Adult treatment panel III), 2001). Additionally, we calculated the Chol/HDL (Conraads et al., 2003), TG/HDL (Oliveri et al., 2024), and LDL/HDL (Di Taranto et al., 2019) ratios to evaluate their relevance to seroprotection from the hepatitis B vaccine.

### 2.4 Covariates

Based on factors known to potentially impact the immune response to the hepatitis B vaccine and serum lipid levels, as outlined in the CDC’s Red Book and previous studies (Christensen et al., 2015; Penina Haber and Schillie, 2023), we selected several covariates, including age, sex, body mass index (BMI), race/ethnicity, family poverty-to-income ratio (PIR), country of birth, and smoking status. BMI was used to categorize participants into normal weight (< 25 kg/m²), overweight (25-29.9 kg/m²), and obese (≥ 30 kg/m²) according to CDC cutoffs for adults (CDC, 2023). Participants were classified as exposed to environmental smoke or active smokers (≥ 10 ng/mL) and nonsmokers (< 10 ng/mL) based on their serum cotinine levels (Andrews et al., 2021).

### 2.5 Statistical analysis

Given the complex multistage probability sampling and oversampling of specific subgroups within the NHANES dataset, we applied data weighting using a “survey design” approach to enhance the accuracy of statistics and ensure they more accurately reflect the true distribution of the U.S. population. We employed the 2-Year Mobile Examination Center Weight for Fasting Subsample (WTSAF2YR) in our weighted analysis, as triglyceride measurements were collected in the fasting subsample. For the final analysis, the sample weight was determined as the average of the “WTSAF2YR” values from seven NHANES survey cycles.

Continuous variables, such as Chol, TG, HDL, and LDL, were categorized based on previous literature (Executive summary of the third report of the national cholesterol education program (NCEP) expert panel on detection, evaluation, and treatment of high blood cholesterol in adults (Adult treatment panel III), 2001). Summary statistics, including survey-weighted means and 95% confidence intervals for continuous variables, as well as unweighted sample sizes and survey-weighted percentages for categorical variables, described the baseline characteristics of the study participants. In the analysis of continuous variables, we utilized the Kruskal-Wallis rank sum test, and for situations involving fewer than ten theoretical count variables, the Fisher’s exact probability test was applied. Categorical data were subjected to *p*-value determination through weighted chi-square analysis (refer to **Table 1**).

**Table 1:** Characteristics of 6530 participants in NHANES data, 2003-2016.

Characteristics	Susceptible to HBV N=3254	Immunity from Hepatitis B Vaccination N=3276	*P* value
	Mean (95% CI) or n(%)	Mean (95% CI) or n(%)	
**Age(years)**	32.6 (31.8,33.4)	29.4 (28.5,30.4)	<0.01
**Age(years)**			<0.01
6-19	1437 (31.5)	1821 (33.5)	
20-39	933 (35.2)	934 (41.0)	
40-59	568 (24.7)	377 (20.3)	
≥ 60	316 (8.6)	144 (5.3)	
**Sex**			<0.01
Male	1603 (48.0)	1418 (41.0)	
Female	1651 (52.0)	1858 (59.0)	
**Race/Ethnicity**			<0.01
Mexican American	681 (10.7)	670 (7.7)	
Other Hispanic	283 (6.2)	223 (4.7)	
Non-Hispanic White	1159 (63.5)	1207 (67.6)	
Non-Hispanic Black	876 (14.8)	868 (12.0)	
Other Race - Including Multi-Racial	255 (5.8)	308 (8.1)	
**Country of Birth**			0.41
U.S.- born	2699 (87.9)	2755 (87.9)	
non-U.S.-born	553 (12.0)	520 (12.0)	
Refused	2 (0.1)	1 (0.0)	
**Family poverty/income ratio**			<0.01
< 1.5	1455 (31.6)	1327 (25.4)	
1.5-3.5	1039 (34.7)	987 (29.7)	
≥ 3.5	760 (33.7)	962 (44.9)	
**Serum Cotinine (ng/mL)**			<0.01
non-smoker (< 10)	2614 (77.6)	2778 (82.1)	
Smoker (≥ 10)	640 (22.4)	498 (17.9)	
**Body Mass Index (kg/m^2^)**			<0.01
Normal weight (< 25)	1460 (41.0)	1854 (51.2)	
Overweight (25-29.9)	802 (25.9)	754 (26.0)	
Obese (≥ 30)	992 (33.1)	668 (22.8)	
**HDL (mg/dL)**	52.1 (51.5,52.8)	55.9 (55.2,56.7)	<0.01
**HDL (mg/dL)**			<0.01
Desirable(≥ 60)	896 (26.0)	1109 (35.0)	
Borderline (40/50-59)	1446 (43.7)	1400 (42.4)	
Low (< 40 for man, < 50 for women)	912 (30.3)	767 (22.6)	
**TG (mg/dL)**	109.0 (105.9,112.1)	100.0 (97.0,103.1)	<0.01
**TG (mg/dL)**			<0.01
Desirable (< 150)	2640 (79.5)	2848 (84.9)	
Borderline (150–199)	333 (10.8)	243 (8.5)	
High (200–499)	281 (9.7)	185 (6.5)	
**LDL (mg/dL)**	105.6 (103.7,107.6)	103.3 (101.7,104.9)	0.07
**LDL (mg/dL)**			0.23
Desirable for high risk (< 70)	500 (12.5)	575 (14.0)	
Desirable (70–99)	1226 (35.4)	1308 (36.2)	
Near optimal (100–129)	899 (29.4)	906 (29.8)	
Borderline (130–159)	434 (15.5)	354 (14.6)	
High (160–189)	151 (5.6)	94 (3.9)	
Very high (≥ 190)	44 (1.6)	39 (1.6)	
**Chol (mg/dL)**	179.5 (177.4,181.6)	179.2 (177.2,181.3)	0.84
**Chol (mg/dL)**			0.39
Desirable(< 200)	2472 (71.8)	2600 (73.3)	
Borderline (200–239)	782 (20.1)	675 (19.8)	
High (≥ 240)	0 (8.1)	1 (6.9)	
**TG/HDL**	2.4 (2.3,2.5)	2.0 (1.9,2.1)	<0.01
**Chol/HDL**	3.7 (3.6,3.7)	3.4 (3.3,3.4)	<0.01
**LDL/HDL**	2.2 (2.1,2.2)	2.0 (1.9,2.0)	<0.01
Hyperlipidemia			<0.01
No	3150 (95.9)	3211 (97.5)	
Yes	104 (4.1)	65 (2.5)	

Chol, total cholesterol; LDL, LDL cholesterol; HDL, HDL cholesterol; TG, triglycerides.

For continuous variables, the data were presented as survey-weighted mean (95% CI). For categorical variables, the data were presented as unweighted sample sizes and survey-weighted percentage.

**Figure 2:**
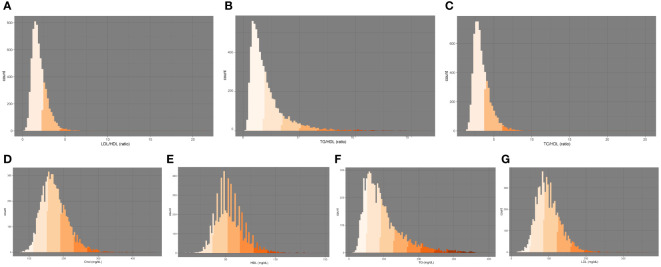
Distribution of serum lipid markers: **(A)** LDL/HDL, **(B)** TG/HDL, **(C)** TC/HDL, **(D)** Chol, **(E)** HDL, **(F)** TG, **(G)** LDL. Chol, total cholesterol; HDL, high-density lipoprotein cholesterol, TG, triglycerides, LDL, low-density lipoprotein cholesterol.

Furthermore, we conducted four statistical tests, namely, the Anderson-Darling normality test, the Cramer-von Mises normality test, the Lilliefors (Kolmogorov-Smirnov) normality test, and the Pearson chi-square normality test (refer to **Supplementary Table S2**). We employed the “ggplot2” R package to create visual representations of the data distribution, as illustrated in **Figure 2**. And we applied a log2 transformation during regression analysis to account for non-normality. To assess the independent impact of lipid concentrations on the response to the HBV vaccine, we utilized multivariable weighted linear regression models. These models included Model I, which was unadjusted for covariates; Model II, adjusted for age, PIR, and sex; and Model III, adjusted for age, race, sex, PIR, serum cotinine, place of birth, and BMI. Covariate selection adhered to internationally recognized practices, introducing or removing variables based on their effect on the regression coefficient of X exceeding 10%.

To evaluate potential nonlinear relationships, we performed smooth curve fitting and segmentation effect analysis. Subsequently, we conducted a subgroup analysis employing stratified multivariate logistic regression and interaction testing to explore stratified relationships between serum lipid levels and HBV vaccine-induced immunity, as well as the interactive effects of covariates on this relationship.

For sensitivity analysis, we categorized HDL, TG, and other lipid-related indexes into quartiles (Q1-Q4), as shown in **Supplementary Table S4**. All statistical analyses were performed using R software (Version 4.2.3), the R package, and EmpowerStats (www.empowerstats.com), with a significance level set at *P* < 0.05. FigDraw was utilized for graphical illustrations.

## 3 Results

### 3.1 Baseline characteristics

The analysis included a total of 6,530 individuals, comprising 3,276 participants with immunity following hepatitis B vaccination and 3,254 individuals susceptible to HBV. **Table 1** outlines the baseline characteristics, highlighting significant differences between those with post-vaccination immunity and those susceptible to HBV concerning age, gender, race, PIR, BMI, smoking status, as well as levels of HDL, TG, TG/HDL ratio, Chol/HDL ratio, LDL/HDL ratio, and the presence of hyperlipidemia.

The seroprotection group was characterized by a higher proportion of females (59.0%), a younger age demographic (age < 40), non-smokers (82.1%), non-Hispanic whites (67.6%), individuals with a PIR ≥ 3.5, higher HDL levels (55.9 mg/dL, 95% CI 55.2-56.7), lower TG levels (100.0 mg/dL, 95% CI 97.0-103.1), a lower TG/HDL ratio (2.0, 95% CI 1.9-2.1), a lower Chol/HDL ratio (3.4, 95% CI 3.3-3.4), a lower LDL/HDL ratio (2.0, 95% CI 1.9-2.0), and a lower prevalence of hyperlipidemia (97.5%).

When examining lipid levels as categorical variables, the seroprotection group demonstrated a higher percentage of individuals with ‘desirable’ levels of HDL and TG compared to the susceptible group. Nevertheless, no significant differences were observed in Chol and LDL levels between these two groups.

### 3.2 The connection between serum lipid levels and immunity from hepatitis B vaccination

To investigate the association between serum lipid levels and HBV vaccination-induced immunity, we developed three weighted logistic regression models, as presented in **Table 2**. In Model 1, where no variables were taken into account, we observed significant association between HDL, TG, LDL/HDL, TG/HDL, and TC/HDL ratios and post-vaccination immunity. Remarkably, even after comprehensive adjustments for all variables, these associations persisted.

**Table 2:** Associations between lipid levels and immunity from Hepatitis B vaccination in NHANES, 2003-2016.

Exposure	Model 1 cOR (95% CI)	Model 2 aOR (95% CI)	Model 3 aOR (95% CI)
HDL (mg/dL)			
Desirable (≥ 60)	Reference	Reference	Reference
Borderline (40/50-59)	0.72*** (0.61-0.85)	0.80* (0.67-0.96)	0.83* (0.69-0.99)
Low (< 40 for man, < 50 for women)	0.55****(0.47-0.66)	0.60****(0.50-0.71)	0.65****(0.56-0.78)
TG (mg/dL)			
Desirable (< 150)	Reference	Reference	Reference
Borderline (150–199)	0.74** (0.61-0.90)	0.86 (0.70-1.06)	0.91 (0.74-1.13)
High (200–499)	0.63*** (0.49-0.82)	0.74* (0.57-0.97)	0.79 (0.60-1.05)
**Log2-TG/HDL**	0.80**** (0.75-0.86)	0.87*** (0.81-0.94)	0.90* (0.84-0.98)
**Log2-Chol/HDL**	0.57**** (0.49-0.67)	0.70*** (0.59-0.83)	0.77** (0.64-0.92)
**Log2-LDL/HDL**	0.70**** (0.63-0.78)	0.80***(0.71-0.90)	0.85** (0.76-0.96)

The reference group for the outcome variable is the population that did not acquire immunity after hepatitis B vaccination.

95% CI, 95% Confidence Interval; OR, Odds Ratio.

model I, unadjusted for covariates; model II, adjusted for age, sex, and PIR; model III, adjusted for age, sex, PIR, serum cotinine, place of birth, race, and BMI.

*P-value < 0.05; ** P-value < 0.01; ***P-value < 0.001; ****P-value < 0.0001.

Specifically, a reduction in HBV vaccination-induced immunity was associated with lower HDL levels, but higher TG, TG/HDL, Chol/HDL, and LDL/HDL ratios. Using HDL levels of ≥ 60 mg/dL as a reference, the ORs for the borderline group (40/50-59 mg/dL) and the low group (<40 mg/dL for men, <50 mg/dL for women) were 0.83 (95% CI 0.69-0.99) and 0.65 (95% CI 0.56-0.78), respectively, indicating a 17% and 35% reduction in serological protection following vaccination. Additionally, for each 1-unit increment in log2-TG/HDL, log2-Chol/HDL, and log2-LDL/HDL ratios, vaccine-induced immunity decreased by 10%, 23%, and 15%, respectively.

### 3.3 Stratified analysis

**Figure 3:**
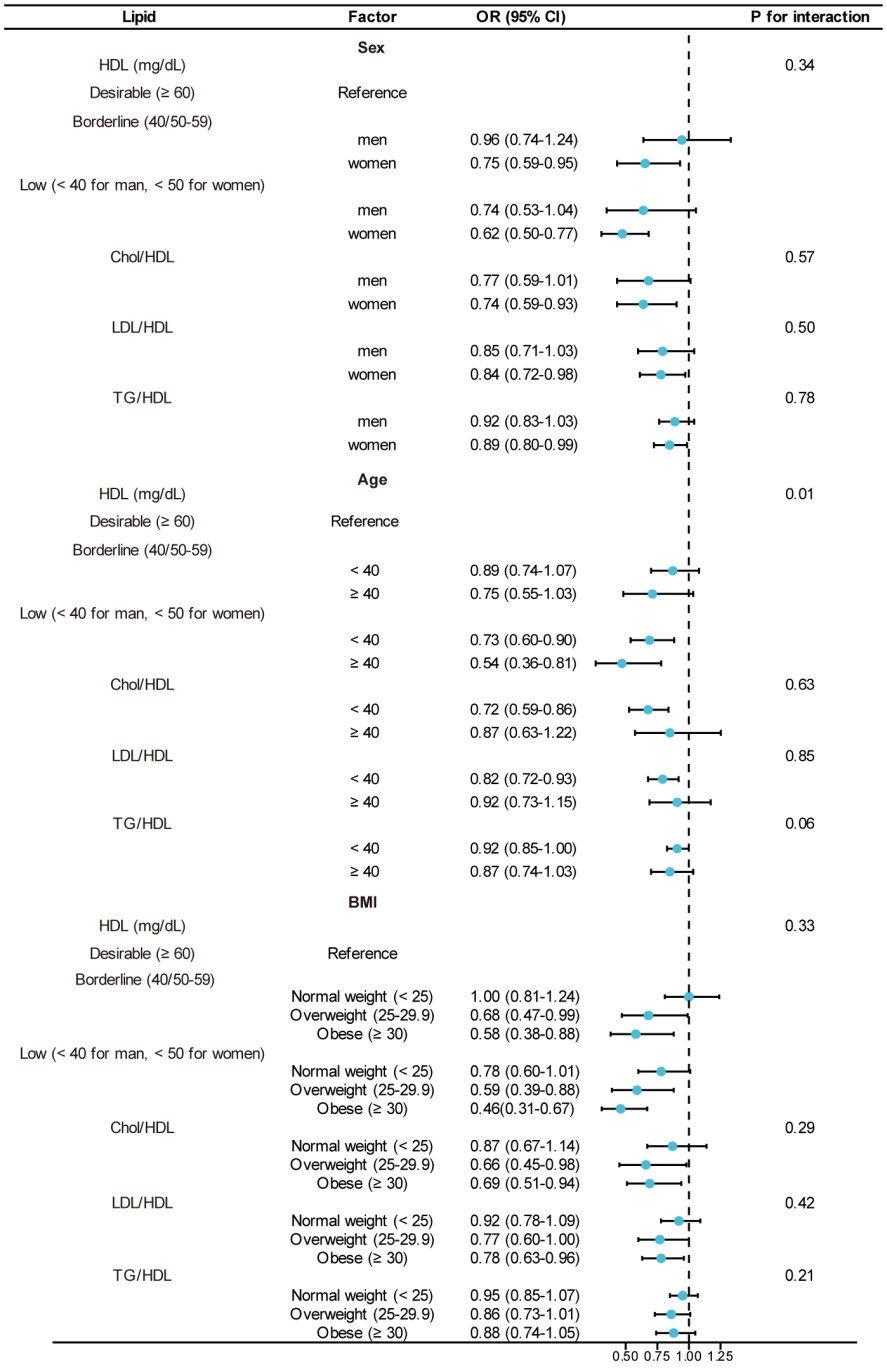
Subgroup analysis for the association between lipid levels and immunity from Hepatitis B vaccination: 95% CI, 95% Confidence Interval; OR, Odds Ratio. Adjusted for age, sex, PIR, serum cotinine, place of birth, race, and BMI. The model was not adjusted for the stratification variable itself.

In the fully adjusted model, we delved deeper into the association between serum lipid concentrations and seroprotection following vaccination within specific subgroups categorized by sex, BMI, and age, as depicted in **Figure 3**. Furthermore, we conducted interaction analyses on the three regression models, considering variables such as age, gender, BMI, smoking, PIR, and race, as detailed in **Supplementary Table S3**.

We observed that these variables did not exhibit significant interactions concerning the association between LDL/HDL, Chol/HDL, and post-HBV vaccination immunity. However, an interaction emerged with smoking and TG/HDL, where individuals in the smoking group experienced a 16% reduction in the likelihood of post-HBV vaccination immunity for each unit increase in TG/HDL.

Additionally, age demonstrated an interaction effect with HDL, underscoring a more pronounced association between HDL and post-HBV vaccination immunity among individuals aged 40 and older.

### 3.4 Nonlinear or linear association between lipid levels and serological protection

**Figure 4:**
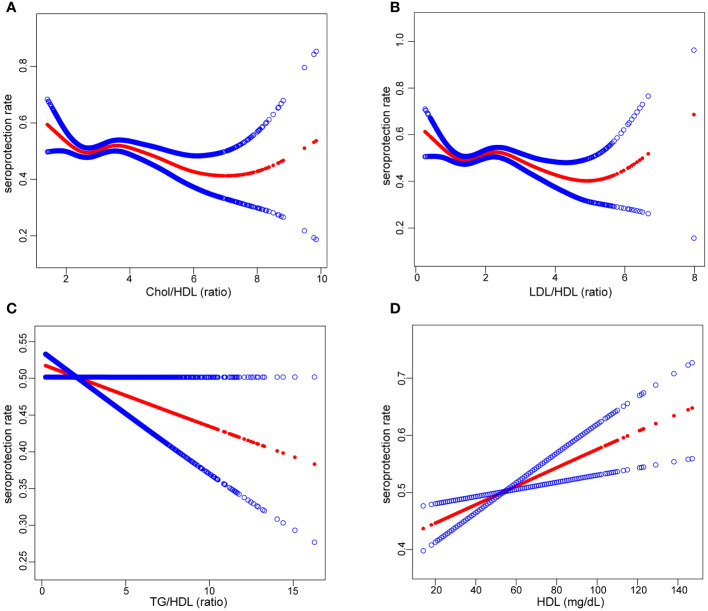
The relationship between serum lipid levels and seroprotection rate: Liner and non-liner association between Chol/HDl **(A)**, LDL/HDL **(B)**, TG/HDL **(C)**, HDl **(D)** and seroprotection rate. The solid red line represents the smooth curve fit between variables. Blue bands represent the 95% confidence interval from the fit. Chol, total cholesterol; HDL, high-density lipoprotein cholesterol, TG, triglycerides, LDL, low-density lipoprotein cholesterol.

In the final phase of our analysis, we implemented smooth curve fitting to examine potential partitioning of the independent variable into multiple intervals, as visualized in **Figure 4**. Additionally, we explored the segmentation effect, employing the saturation threshold effect, which is outlined in **Table 3**.

**Table 3:** Treshold effect analysis of Lipid-related Index on Immunity from Hepatitis B Vaccination using Segmented regression models.

	Crude OR (95% CI)	*P*		Adjusted OR (95% CI)	*P*
**LDL/HDL**		0.19	**LDL/HDL**		0.04
LDL/HDL < 2.50	0.82*** (0.74, 0.91)		LDL/HDL < 2.54	1.02 (0.92, 1.14)	
LDL/HDL ≥ 2.50	0.72*** (0.63, 0.82)		LDL/HDL ≥ 2.54	0.82** (0.72, 0.95)	
**Chol/HDL**		0.54	**Chol/HDL**		0.07
Chol/HDL < 3.90	0.82*** (0.75, 0.90)		Chol/HDL < 2.29	0.55* (0.30, 0.99)	
Chol/HDL ≥ 3.90	0.78*** (0.71, 0.86)		Chol/HDL ≥ 2.29	0.96 (0.90, 1.01)	
**TG/HDL**		0.04	**TG/HDL**		0.08
TG/HDL < 2.50	0.82*** (0.75, 0.88)		TG/HDL < 1.14	1.25 (0.94, 1.65)	
TG/HDL ≥ 2.50	0.92*** (0.88, 0.96)		TG/HDL ≥ 1.14	0.95* (0.92, 0.99)	

The reference group for the outcome variable is the population that did not acquire immunity after hepatitis B vaccination.

Crude OR: unadjusted for covariates.

Adjusted OR: adjusted for age, sex, PIR, serum cotinine, place of birth, race, and BMI.

95% CI, 95% Confidence Interval; OR, Odds Ratio.

*P-value < 0.05; **P-value < 0.01; ***P-value < 0.001.

Notably, our findings unveiled a relationship between HDL concentration and an increase in seroprotection. Conversely, TG/HDL and Chol/HDL displayed a linear association with a decrease in seroprotection. Furthermore, we identified negative segmental linear effects in the case of LDL/HDL. Even after adjusting for variables such as sex, age, race, PIR, BMI, and place of birth, LDL/HDL levels ≥ 2.54 exhibited a significant association with a decrease in seroprotection, with an OR of 0.82 (95% CI 0.72-0.95).

### 3.5 Sensitivity analysis

In our sensitivity analysis, serum lipid concentrations were discretized from continuous variables into categorical variables (Q1-Q4). The outcomes of this sensitivity analysis were consistent with the results obtained from the weighted linear regression models. Notably, we observed that as HDL levels increased, the impact on seroprotection following HBV vaccination became progressively more significant. These sensitivity analysis findings revealed that the segment of the American population with higher serum lipid concentrations exhibited a stronger association with post-HBV vaccination immunity compared to the segment with lower serum lipid concentrations. Additional details regarding the sensitivity analysis can be found in **Supplementary Table S4**.

## 4 Discussion

This study delved into the potential association between serum lipid levels and immunity following HBV vaccination, utilizing data from the NHANES database spanning from 2003 to 2016, representative of the US population. Our analysis of 6,530 participants revealed distinct patterns: individuals with seroprotection from vaccination were more likely to be female, younger, and nonsmokers, aligning with previous research (Alavian et al., 2008; Deng et al., 2022; Di Lello et al., 2022; Fonzo et al., 2022; Lian and Morrish, 2022). Notably, our study was the first to establish a significant association between elevated HDL levels and reduced TG, TG/HDL, Chol/HDL, and LDL/HDL levels with seroprotection from HBV vaccination. Even after adjusting for pertinent covariates, these lipid levels remained independent risk factors for HBV vaccination immunity. Additionally, among participants aged 40 and older, heightened HDL levels were associated with a significant enhancement in seroprotection. Furthermore, our research unveiled that when LDL/HDL exceeded 2.54, the likelihood of HBV vaccination-induced immunity significantly declined.

Recent years have seen clinical database analysis significantly advancing disease diagnosis and prognosis, providing fresh insights into disease diagnosis and treatment (Zhang J. et al., 2023; Ren et al., 2023; Chi et al., 2023; Zhang P. et al., 2023; Zhang S. et al., 2023; Zhang et al., 2024; Zhang D. et al., 2023). Among these, several studies have examined the relationship between inflammatory markers and lipid levels. For instance, Xiao et al. reported a linear negative association between the systemic immune inflammation (SII) index and TG (Xiao et al., 2023). In contrast, Mahemuti et al. found a notable positive correlation between SII and hyperlipidemia (Mahemuti et al., 2023). In a separate study involving uterine leiomyoma patients, the neutrophil-lymphocyte ratio and SII exhibited a significant positive correlation with TG, while the monocyte-lymphocyte ratio demonstrated a notable negative correlation with TG (Duan et al., 2023). Discrepancies in these study outcomes may arise from variations in the studied populations and the utilization of different indicators. Moreover, Li et al. identified an inverse relationship between thyroglobulin antibody positivity and HDL levels, as well as a direct association with LDL levels in the general population with normal thyrotropin levels. This relationship was also influenced by gender (Li et al., 2021). These prior findings offer potential support for the observations presented in this study. However, it is important to note that the association between lipid levels and immunity remains a topic of ongoing debate.

The underlying mechanisms governing the association between lipid levels and immunity remain elusive. For instance, Kochumon et al. have demonstrated the pivotal role of IL-23 in the pathogenesis of inflammation induced by elevated low-density lipoprotein cholesterol (Kochumon et al., 2022). Furthermore, lipoxins and metabolites of omega-3 fatty acids have been linked to inflammation resolution (Bäck et al., 2015). In a review by Nancy R. Webb, it was proposed that high-density lipoprotein may contribute to the *in vivo* regulation of serum amyloid A, thereby influencing the inflammatory response (Webb, 2021). Additionally, lipid mediators like leukotrienes and prostaglandins have been shown to modulate mast cell (MC) functions (Hagemann et al., 2019). Abnormal accumulation of endogenous lipids or their oxidation products can activate NLRP3, subsequently triggering inflammatory responses (Liang et al., 2021). Short-chain fatty acids have been reported to impact immune function through the stimulation of GPR43 or GPR41, leading to increased regulatory T cell numbers and enhanced function, while also reducing inflammatory cytokines (Tan et al., 2023). Schümann et al. reviewed ApoE (and potentially other apolipoproteins)-mediated lipid antigen transport, revealing its critical role in tumor immune surveillance and offering new perspectives for immunotherapy and vaccines (Schümann and De Libero, 2006). In summary, the interplay between lipid levels and immunity involves intricate mechanisms that warrant further investigation.

The present study unveils a novel association between serum lipid levels and immunity from HBV vaccination. Specifically, HDL cholesterol levels exhibit an association with an increase in seroprotection, while TG/HDL, Chol/HDL, and LDL/HDL levels display associations with a decrease in seroprotection. Our study boasts several merits. Firstly, the extensive dataset sourced from NHANES bolsters the reliability and applicability of our findings. Secondly, we meticulously employed appropriate methodologies to mitigate the influence of confounding variables. Furthermore, our results underwent weighting to reduce the potential bias stemming from population selection and augment overall representativeness.

However, it is crucial to acknowledge several limitations. Firstly, while the NHANES sample is extensive and representative of the US population, it may not comprehensively reflect other demographics or geographic regions. Secondly, despite our efforts to control for pertinent covariates, we cannot entirely dismiss the impact of unmeasured confounding factors on the observed associations. Thirdly, due to the natural exposure and time-dependent waning of serological protection following vaccine administration, there exists a potential for inherent bias in the outcomes of this study (31,Van Damme et al., 2017; Kushner et al., 2020). Lastly, this study, based on real-world data from a large population sample, sheds light on the potential association between serum lipid levels and the serological protection conferred by hepatitis B vaccination. However, further prospective research or animal-level verification is still needed to confirm the causal relationship between serum lipid levels and serological protection following hepatitis B vaccination.

Notwithstanding these constraints, our findings provide valuable insights into the prospective role of lipid metabolism in HBV vaccine response, potentially leading to enhanced vaccination strategies and more robust protection against HBV-related diseases.

## 5 Conclusion

Through the utilization of weighted logistic regression models and saturation threshold effect analysis, we highlight a distinctive relationship between lipid levels and immunity post HBV vaccination. Our findings underscore the positive association of HDL with seroprotection, while indicating negative associations of TG/HDL, LDL/HDL, and Chol/HDL with seroprotection. To substantiate potential causal links within our results, comprehensive prospective studies are imperative.

## Data availability statement

The datasets presented in this study can be found in online repositories. The names of the repository/repositories and accession number(s) can be found in the article/**Supplementary Material**.

## Author contributions

SC: Conceptualization, Writing – review & editing. QY: Writing – original draft, Writing – review & editing. BL: Methodology, Visualization, Writing – review & editing. TL: Methodology, Visualization, Writing – review & editing. XW: Data curation, Software, Writing – review & editing. BD: Data curation, Software, Writing – review & editing. CW: Resources, Supervision, Writing – review & editing.
