# Supplementary material for: Exploring blood lipids-immunity associations following HBV vaccination: evidence from a large cross-sectional study
Source: Front Cell Infect Microbiol. 2024 Mar 8;14:1369661. doi: 10.3389/fcimb.2024.1369661 (PMC10959126; doi:10.3389/fcimb.2024.1369661)
Supplement: Supplementary file 1 [file DataSheet_1.pdf]

Supplementary Table S1. Distribution of Lipid-related Index among American.

Index	N	Mean	Standard	Min	25% digits	50% digits	75% digits	Max
TG/HDL	6530	2.07	1.69	0.23	0.98	1.55	2.53	16.26
TC/HDL	6530	3.39	1.12	1.41	2.62	3.15	3.94	25.1
LDL/HDL	6530	1.98	0.89	0.27	1.36	1.80	2.43	21.3

Supplementary Table S2. Normality test of Lipid-related Index among American.

	Method	Statistic	P
TG/HDL			
	Anderson-Darling normality test	392.61	<0.01
	Cramer-von Mises normality test	69.65	<0.01
	Lilliefors (Kolmogorov-Smirnov) normality test	0.16	<0.01
	Pearson chi-square normality test	4473.68	<0.01
TC/HDL			<0.01
	Anderson-Darling normality test	139.35	<0.01
	Cramer-von Mises normality test	23.33	<0.01
	Lilliefors (Kolmogorov-Smirnov) normality test	0.098	<0.01
	Pearson chi-square normality test	1483.39	<0.01
LDL/HDL			<0.01
	Anderson-Darling normality test	114.55	<0.01
	Cramer-von Mises normality test	19.14	<0.01
	Lilliefors (Kolmogorov-Smirnov) normality test	0.09	<0.01
	Pearson chi-square normality test	1197.94	<0.01

Supplementary Table S3 Interaction analysis of the association of lipid levels and immunity from Hepatitis B vaccination

Index			Model 1		Model 2		Model 3	
			OR (95% CI)	P for interaction	OR (95% CI)	P for interaction	OR (95% CI)	P for interaction
Log2-LDL/HDL	Age	<40	0.77 (0.70, 0.85)	0.9347	0.79 (0.72, 0.87)	0.7805	0.88 (0.80, 0.98)	0.8599
		⩾40	0.77 (0.64, 0.91)		0.81 (0.68, 0.97)		0.87 (0.72, 1.04)	
	Sex	male	0.74 (0.66, 0.83)	0.6991	0.86 (0.77, 0.97)	0.4774	0.92 (0.81, 1.04)	0.4989
		female	0.72 (0.64, 0.80)		0.81 (0.72, 0.92)		0.87 (0.77, 0.98)	
	BMI	<25	0.88 (0.78, 1.00)		0.95 (0.84, 1.08)		0.94 (0.83, 1.07)	
		25-30	0.70 (0.59, 0.83)	0.0986	0.82 (0.69, 0.98)	0.4030	0.82 (0.69, 0.97)	0.4236
		⩾30	0.83 (0.70, 0.98)		0.89 (0.75, 1.06)		0.89 (0.75, 1.06)	

	Smoking	No	0.72 (0.66, 0.79)	0.6385	0.84 (0.76, 0.92)	0.7913	0.89 (0.81, 0.99)	0.8635
		Yes	0.76 (0.63, 0.90)		0.86 (0.72, 1.03)		0.91 (0.76, 1.09)	
	PIR	< 1.5	0.71 (0.63, 0.80)	0.9899	0.83 (0.73, 0.94)	0.8679	0.89 (0.78, 1.01)	0.8520
		1.5-3.5	0.71 (0.62, 0.83)		0.83 (0.71, 0.96)		0.87 (0.75, 1.02)	
		⩾ 3.5	0.72 (0.62, 0.84)		0.87 (0.74, 1.02)		0.93 (0.79, 1.09)	
	Race /Ethnicity	Mexican American	0.69 (0.58, 0.84)	0.2475	0.81 (0.67, 0.97)	0.3771	0.86 (0.71, 1.05)	0.3481
		Other Hispanic	0.51 (0.38, 0.70)		0.63 (0.46, 0.87)		0.66 (0.48, 0.91)	
		Non-Hispanic White	0.72 (0.63, 0.82)		0.87 (0.76, 1.00)		0.94 (0.82, 1.08)	
		Non-Hispanic Black	0.75 (0.65, 0.88		0.84 (0.72, 0.98)		0.90 (0.77, 1.05)	
		Other Race - Including Multi-Racial	0.79 (0.59, 1.05)		0.94 (0.70, 1.26)		0.96 (0.72, 1.29)	
Log2-TG/ HDL	Age	<40	0.88 (0.83, 0.93)	0.0350	0.89 (0.84, 0.95)	0.1193	0.96 (0.90, 1.03)	0.0665
		⩾40	0.77 (0.69, 0.86)		0.81 (0.72, 0.90)		0.86 (0.76, 0.96)	
	Sex	male	0.83 (0.77, 0.89)	0.8722	0.91 (0.85, 0.98)	0.7530	0.95 (0.88, 1.03)	0.7795
		female	0.82 (0.76, 0.88)		0.90 (0.83, 0.96)		0.94 (0.87, 1.01)	
	BMI	<25	0.94 (0.86, 1.02)	0.0811	0.99 (0.91, 1.07)	0.2005	0.99 (0.91, 1.07)	0.2055
		25-30	0.81 (0.73, 0.90)		0.87 (0.79, 0.97)		0.87 (0.79, 0.97)	
		⩾30	0.89 (0.80, 0.99)		0.96 (0.86, 1.07)		0.95 (0.85, 1.06)	
	Smoking	No	0.84 (0.80, 0.89)	0.0760	0.93 (0.87, 0.98)	0.0557	0.97 (0.91, 1.03)	0.0434
		Yes	0.75 (0.66, 0.84)		0.81 (0.72, 0.92)		0.84 (0.74, 0.96)	
	PIR	< 1.5	0.79 (0.73, 0.85)	0.4062	0.87 (0.80, 0.94)	0.3822	0.91 (0.84, 0.99)	0.4179
		1.5-3.5	0.85 (0.78, 0.93)		0.94 (0.86, 1.03)		0.99 (0.89, 1.08)	
		⩾ 3.5	0.83 (0.75,		0.91 (0.83, 1.01)		0.95 (0.86,	

			0.91)				1.06)	
	Race /Ethnicity	Mexican American	0.84 (0.75, 0.94)		0.93 (0.83, 1.04)		0.97 (0.86, 1.09)	
		Other Hispanic	0.79 (0.66, 0.95)		0.89 (0.74, 1.07)		0.93 (0.77, 1.12)	
		Non-Hispanic White	0.80 (0.73, 0.87)	0.5423	0.90 (0.82, 0.98)	0.7256	0.95 (0.87, 1.04)	0.6161
		Non-Hispanic Black	0.83 (0.75, 0.92)		0.91 (0.81, 1.01)		0.96 (0.86, 1.07)	
		Other Race - Including Multi-Racial	0.70 (0.58, 0.84)		0.79 (0.66, 0.96)		0.82 (0.68, 0.99)	
Log2-Chol /HDL	Age	<40	0.67 (0.58, 0.76)	0.6071	0.69 (0.60, 0.79)	0.9726	0.82 (0.71, 0.96)	0.6347
		≥40	0.62 (0.48, 0.80)		0.69 (0.53, 0.89)		0.77 (0.59, 0.99)	
	Sex	male	0.61 (0.52, 0.72)	0.6618	0.78 (0.66, 0.92)	0.5350	0.86 (0.72, 1.03)	0.5721
		female	0.58 (0.49, 0.69)		0.72 (0.61, 0.86)		0.80 (0.67, 0.96)	
	BMI	<25	0.81 (0.67, 0.99)		0.93 (0.76, 1.14)		0.92 (0.75, 1.13)	
		25-30	0.57 (0.45, 0.73)	0.0838	0.72 (0.56, 0.92)	0.2714	0.71 (0.56, 0.92)	0.2871
		≥30	0.72 (0.57, 0.91)		0.83 (0.65, 1.06)		0.82 (0.64, 1.06)	
	Smoking	No	0.60 (0.53, 0.69)	0.8372	0.77 (0.67, 0.88)	0.6244	0.85 (0.73, 0.98)	0.5346
		Yes	0.59 (0.45, 0.76)		0.71 (0.55, 0.93)		0.77 (0.59, 1.01)	
	PIR	< 1.5	0.56 (0.47, 0.67)		0.72 (0.60, 0.86)		0.80 (0.66, 0.97)	
		1.5-3.5	0.60 (0.48, 0.74)	0.8495	0.76 (0.61, 0.94)	0.7651	0.83 (0.66, 1.04)	0.7897
		≥ 3.5	0.60 (0.48, 0.75)		0.80 (0.63, 1.00)		0.88 (0.70, 1.12)	
	Race /Ethnicity	Mexican American	0.58 (0.45, 0.76)		0.74 (0.56, 0.96)		0.81 (0.62, 1.07)	
		Other Hispanic	0.39 (0.25, 0.61)		0.54 (0.35, 0.84)		0.58 (0.37, 0.91)	
				0.4939		0.6297		0.5480
		Non-Hispanic White	0.59 (0.49, 0.71)		0.79 (0.65, 0.96)		0.89 (0.73, 1.09)	
		Non-Hispanic Black	0.61 (0.48, 0.77)		0.74 (0.58, 0.94)		0.83 (0.65, 1.07)	

		Other Race - Including Multi-Racial	0.59 (0.38, 0.90)		0.80 (0.51, 1.23)		0.84 (0.54, 1.30)	
HDL	Age	<40	1.01 (1.01, 1.02)	0.0201	1.01 (1.00, 1.01)	0.0379	1.00 (1.00, 1.01)	0.0296
		⩾40	1.02 (1.01, 1.03)		1.02 (1.01, 1.02)		1.01 (1.01, 1.02)	
	Sex	male	1.01 (1.01, 1.02)	0.8878	1.01 (1.00, 1.01)	0.2040	1.00 (1.00, 1.01)	0.2124
		female	1.01 (1.01, 1.02)		1.01 (1.01, 1.02)		1.01 (1.00, 1.01)	
	BMI	<25	1.01 (1.00, 1.01)	0.4278	1.01 (1.00, 1.01)	0.5664	1.01 (1.00, 1.01)	0.5951
		25-30	1.01 (1.00, 1.02)		1.01 (1.00, 1.02)		1.01 (1.00, 1.02)	
		⩾30	1.01 (1.00, 1.02)		1.01 (1.00, 1.02)		1.01 (1.00, 1.02)	
	Smoking	No	1.01 (1.01, 1.02)	0.3674	1.01 (1.01, 1.01)	0.5200	1.01 (1.00, 1.01)	0.4451
		Yes	1.02 (1.01, 1.02)		1.01 (1.00, 1.02)		1.01 (1.00, 1.02)	
	PIR	< 1.5	1.01 (1.01, 1.02)	0.1266	1.01 (1.00, 1.01)	0.0526	1.01 (1.00, 1.01)	0.0394
		1.5-3.5	1.01 (1.00, 1.01)		1.01 (1.00, 1.01)		1.00 (1.00, 1.01)	
		⩾ 3.5	1.02 (1.01, 1.02)		1.02 (1.01, 1.02)		1.02 (1.01, 1.02)	
	Race /Ethnicity	Mexican American	1.00 (1.00, 1.01)	0.0671	1.00 (0.99, 1.01)	0.1387	1.00 (0.99, 1.01)	0.1352
		Other Hispanic	1.02 (1.00, 1.03)		1.01 (1.00, 1.03)		1.01 (1.00, 1.02)	
		Non-Hispanic White	1.02 (1.01, 1.02)		1.01 (1.01, 1.02)		1.01 (1.01, 1.02)	
		Non-Hispanic Black	1.01 (1.00, 1.02)		1.01 (1.00, 1.02)		1.01 (1.00, 1.01)	
		Other Race - Including Multi-Racial	1.02 (1.01, 1.04)		1.02 (1.00, 1.03)		1.02 (1.00, 1.03)	

Supplementary Table S4. Sensitivity analysis.

Exposure	Model 1		Model 2		Model 3	
	OR (95% CI)	P	OR (95% CI)	P	OR (95% CI)	P
HDL (mg/dL)						

Q1	Reference		Reference		Reference	
Q2	1.18 (1.02, 1.36)	0.02	1.09 (0.95, 1.26)	0.23	1.04 (0.89, 1.20)	0.64
Q3	1.42 (1.24, 1.64)	<0.01	1.27 (1.10, 1.46)	<0.01	1.16 (1.00, 1.34)	0.06
Q4	1.57 (1.37, 1.81)	<0.01	1.39 (1.20, 1.61)	<0.01	1.24 (1.06, 1.45)	<0.01
**P for Trend**	<0.01		<0.01		0.01	
**TG (mg/dL)**						
Q1	Reference		Reference		Reference	
Q2	1.03 (0.89, 1.18)	0.71	1.08 (0.94, 1.24)	0.29	1.12 (0.97, 1.29)	0.12
Q3	0.98 (0.86, 1.13)	0.82	1.14 (0.99, 1.32)	0.07	1.22 (1.05, 1.41)	<0.01
Q4	0.63 (0.55, 0.72)	<0.01	0.82 (0.71, 0.95)	<0.01	0.92 (0.78, 1.07)	0.28
**P for Trend**	<0.01		0.01		0.08	
**TG/HDL**						
Q1	Reference		Reference		Reference	
Q2	0.99 (0.87, 1.14)	0.94	1.04 (0.90, 1.20)	0.57	1.08 (0.94, 1.25)	0.27
Q3	0.83 (0.72, 0.95)	0.01	0.94 (0.82, 1.09)	0.42	1.01 (0.87, 1.17)	0.87
Q4	0.62 (0.54, 0.71)	<0.01	0.80 (0.70, 0.93)	<0.01	0.91 (0.78, 1.07)	0.25
**P for Trend**	<0.01		0.01		0.08	
**Chol/HDL**						
Q1	Reference		Reference		Reference	
Q2	0.87 (0.75, 0.99)	0.04	0.91 (0.79, 1.04)	0.17	0.92 (0.80, 1.06)	0.25
Q3	0.84 (0.73, 0.97)	0.02	0.96 (0.83, 1.11)	0.58	1.03 (0.89, 1.19)	0.68
Q4	0.55 (0.48, 0.63)	<0.01	0.73 (0.63, 0.85)	<0.01	0.82 (0.70, 0.97)	0.02
**P for Trend**	<0.01		<0.01		0.03	
**LDL/HDL**						
Q1	Reference		Reference		Reference	
Q2	0.91 (0.79, 1.04)	0.17	0.94 (0.82, 1.08)	0.41	0.96 (0.83, 1.10)	0.56
Q3	0.86 (0.75, 0.99)	0.04	0.97 (0.84, 1.12)	0.68	1.03 (0.89, 1.19)	0.67
Q4	0.62 (0.54, 0.71)	<0.01	0.81 (0.70, 0.93)	<0.01	0.91 (0.78, 1.06)	0.21
**P for Trend**	<0.01		0.01		0.29	
